# Overview of the Distribution, Habitat Association and Impact of Exotic Ants on Native Ant Communities in New Caledonia

**DOI:** 10.1371/journal.pone.0067245

**Published:** 2013-06-26

**Authors:** Maïa Berman, Alan N. Andersen, Christelle Hély, Cédric Gaucherel

**Affiliations:** 1 Ecosystem Sciences, Commonwealth Scientific and Industrial Research Organisation, Winnellie, Northern Territory, Australia; 2 Research Institute for the Environment and Livelihoods, Charles Darwin University, Casuarina, Northern Territory, Australia; 3 Unité Mixte de Recherche 0931 (botAnique et bioinforMatique de l’Architecture des Plantes), Institut National de la Recherche Agronomique, Université de Montpellier II, Montpellier, France; 4 Unité Mixte de Recherche 5059 (Centre de Bio-Archéologie et Ecologie), Ecole Pratique des Hautes Etudes, Laboratoire Paléoenvironnements et Chronoécologie, Montpellier, France; 5 Unités Mixtes des Instituts Français de Recherche à l'Etranger 21, Institut Français de Pondicherry, Pondicherry, India; Consiglio Nazionale delle Ricerche (CNR), Italy

## Abstract

Ants are among the most ubiquitous and harmful invaders worldwide, but there are few regional studies of their relationships with habitat and native ant communities. New Caledonia has a unique and diverse ant fauna that is threatened by exotic ants, but broad-scale patterns of exotic and native ant community composition in relation to habitat remain poorly documented. We conducted a systematic baiting survey of 56 sites representing the main New Caledonian habitat types: rainforest on ultramafic soils (15 sites), rainforest on volcano-sedimentary soils (13), maquis shrubland (15), *Melaleuca*-dominated savannas (11) and *Acacia spirorbis* thickets (2). We collected a total of 49 species, 13 of which were exotic. Only five sites were free of exotic species, and these were all rainforest. The five most abundant exotic species differed in their habitat association, with *Pheidole megacephala* associated with rainforests, *Brachymyrmex* cf. *obscurior* with savanna, and *Wasmannia auropunctata* and *Nylanderia vaga* present in most habitats. *Anoplolepis gracilipes* occurred primarily in maquis-shrubland, which contrasts with its rainforest affinity elsewhere. Multivariate analysis of overall ant species composition showed strong differentiation of sites according to the distribution of exotic species, and these patterns were maintained at the genus and functional group levels. Native ant composition differed at invaded versus uninvaded rainforest sites, in the absence of differences in habitat variables. Generalised Myrmicinae and Forest Opportunists were particularly affected by invasion. There was a strong negative relationship between the abundance of *W. auropunctata* and native ant abundance and richness. This emphasizes that, in addition to dominating many ant communities numerically, some exotic species, and in particular *W. auropunctata*, have a marked impact on native ant communities.

## Introduction

Ants are among the most ubiquitous and harmful invaders [1], with exotic species distributed worldwide as a result of human activities. Although their ability to disrupt native ecosystems has been widely reported [2]–[5], their presence and impact is often highly dependent on their habitat preference. For example, in California the Argentine ant *Linepithema humile* strongly prefers moist habitats [6], and in monsoonal northern Australia the African big-headed ant *Pheidole megacephala* strongly favors rainforest over savanna [7]. However, there have been few regional studies of the relationship between habitat, exotic ants and native ant communities (e.g. Hill et al. [8]).

Like most Pacific islands [9], the exceptionally biodiverse New Caledonia is a major recipient of exotic ant species. Of Gondwanan origin and with a land area of 18,500 km^2^, this island is also one of the world’s earliest recognized biodiversity hotspots [10]. Its complex biogeographical history [11] and contrasting substrates have led to spectacular radiations and endemism of plants [12], lizards [13], [14] and various invertebrate groups [15], [16]. The ant fauna is no exception, containing diverse radiations of both Gondwanan (e.g. *Rhytidoponera*, *Monomorium*) and Indo-Malayan (e.g. *Lordomyrma*, *Vollenhovia*, *Discothyrea*) taxa [17]–[20]. Unfortunately, this fauna is seriously threatened by habitat loss and invasive ants [17], [21], [22]. Since the arrival of Melanesians approximately 3,500 years ago [23], anthropogenic disturbances such as fire have resulted in approximately half of New Caledonia’s original rainforest vegetation [12] being converted to maquis shrubland on ultramafic soils [24], [25], and to savannas dominated by non-endemic species on volcano-sedimentary soils [26]. Dry sclerophyll forest, which once occupied most of the island’s west coast, now covers only 1% of its original area [12], being replaced by *Acacia spirorbis* (gaiac) thicket [27]. Its exotic ant fauna of at least 26 species [28] includes three of the world’s 100 worst invasive species [1]: *Anoplolepis gracilipes*, present since the early European colonization [29]; *Pheidole megacephala* first reported in 1960 [30]; and *Wasmannia auropunctata*, present since 1972 [31]. These species are known to be able to spread into undisturbed habitats throughout their introduced range [2], [7], [22], causing cascading ecological impacts [2], including dramatic effects on local ant faunas [3].

Because of its fast spread and nuisance for humans *via* its painful sting, most invasive ant research in New Caledonia has focussed on *Wasmannia auropunctata* (originating from central and south America), with a particular emphasis on its behavioural dominance [28], [32]–[34] and particular reproductive biology [35], [36]. The broader distribution of exotic ants on New Caledonia, and their associations with different habitats and effects on native ant communities, have been poorly documented. Here we describe broad-scale patterns of exotic and native ant community composition in relation to habitat, at multiple levels of organisation (species, genus, functional groups).

Our study has three aims: (1) To document the distribution of exotic ant species in New Caledonia in relation to different habitat types and key habitat variables; (2) To assess the extent to which invasive species appear to have modified native ant communities; and (3) To identify particularly sensitive native species and functional groups that are most impacted, and which could thus be used as early indicators of invasion.

## Materials and Methods

### Study Sites

**Figure 1:**
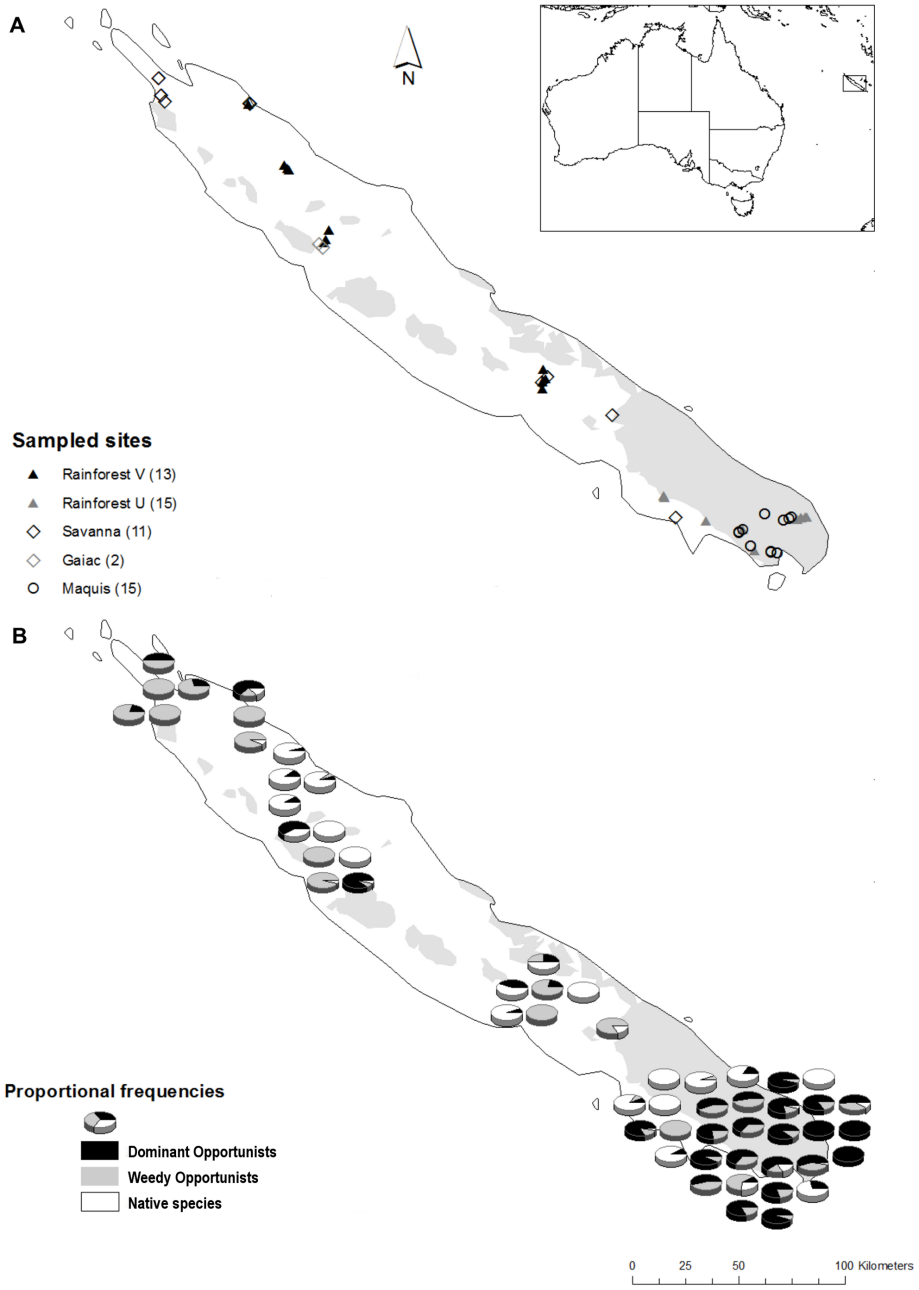
Distribution maps of sampling sites and species collected in New Caledonia. Location of sampling sites in relation to habitat and substrate type (white areas: volcano-sedimentary V; shaded areas: ultramafic U) (a), and proportional frequency (as measured by occurrence in traps) of native ant species, Dominant Opportunists, and exotic Weedy Opportunists (b).

The New Caledonian archipelago (21°30′ S, 165°30′ E) is located approximately 1,200 km east of north-eastern Australia (Figure 1a). Annual rainfall averages 1,058 mm in the coastal capital city Noumea, but can exceed 2,500 mm in inland mountainous regions, with most rainfall occurring between January and June. Mean daily temperatures range from a minimum of 20.1°C in August to a maximum of 26.4°C in February (www.meteo.nc).

Ant sampling was conducted at 56 sites (Table S1; Fig. 1a) representing the five major vegetation types: rainforests on ultramafic soils (15 sites; FUM), rainforests on volcano-sedimentary soils (13; FVS), maquis-shrubland (15; MAQ), anthropogenic savannas dominated by *Melaleuca quinquinervia* (11; SAV), and gaiac thickets (2; GAI). The number of sites per vegetation type was roughly proportional to their respective contribution to the total land surface of New Caledonia.

### Ethic Statement

For each site, sampling permission had been obtained either from the respective Provincial Environmental Departments for natural reserves and other public lands, or from the respective tribes’ committees in the case of sites located on tribal land (Table S1).

### Ant Sampling

Ants were sampled using a simplified baiting protocol, designed to provide a broad overview of the local ant fauna based on standardised sampling, rather than to build a comprehensive species list. Sampling occurred during October 2010, and involved the use of tuna-baited plastic tubes (5 cm×1.5 cm×1.5 cm) placed horizontally on the ground, with the opening flush with the soil surface. At each site, 20 traps were set out in two rows of ten, with 5 m spacing between traps and 20 m between rows. Traps were collected after 2 h, filled with 100% ethanol, capped and returned to the laboratory for processing. Our study is potentially confounded by the fact that ant invasion is still occurring on New Caledonia, with many sites that are remote from anthropogenic disturbance not yet having an ‘opportunity’ for invasion. To control for this to some extent, all our rainforest sites were located within approx. 100 m of an access road or a forest edge.

### Habitat Variables

At each site, we measured habitat variables in order to identify environmental correlates of the distribution of invasive ant species. We also wanted to ensure that invaded sites were not inherently different from uninvaded ones, so that differences in native species composition could be attributed to invasion rather than habitat variation. We quantified five habitat variables that we considered likely to influence ant communities based on the literature [37], [38]: percent cover of litter, grass, shrubs and canopy, and mean litter depth. Percent cover of canopy was estimated using the point-centered quadrant method [39], [40]: at each vertex of a 30 m sided equilateral triangle, the projection surface of the crown of the closest dominant and dominated overstory tree in each of four quadrant was measured (24 tree crowns per sites). All other measurements were made from six 1 m^2^ quadrats placed at 15 m intervals along the triangle sides, either visually, for the cover measurements, or with the use of a ruler at 3 random points in each quadrat for the litter depth.

## Analysis

### Ant Identification and Functional Group Classification

Ants were identified to genus following Bolton [41] and LaPolla et al. [42], and species were named, where possible, using a checklist of ants from New Caledonia [19] and subsequent revisions [20], [43]. However, as most species could not be identified to species level, they were assigned codes within a genus (sp. A, sp. B, etc.) that apply only to this study. Voucher specimens of all species are located at the CSIRO Tropical Ecosystems Research Centre in Darwin. Species were classified as either native or exotic following Jourdan [17]. Where uncertainty existed (e.g. *Pheidole* cf. *oceanica*, and species of *Solenopsis*, *Ochetellus* and *Odontomachus*), we considered them to be native.

We classified species into global functional groups according to responses to environmental stress and disturbance, based on Andersen [44]. These functional groups have proven useful in a wide range of habitats throughout the world [45]–[48], but we modified the Opportunist category to reflect our focus on invasive species (Table S2). We identified three groups of Opportunists: behaviourally dominant exotic species (*W. auropunctata*, *A. gracilipes* and *P. megacephala*; ‘Dominant Opportunists’); behaviourally submissive, mostly exotic species (*Brachymyrmex* cf. *obscurior*, *Odontomachus* cf. *simillimus*; ‘Weedy Opportunists’); and native rainforest opportunists (species of *Leptomyrmex*, *Rhytidoponera* and *Paraparatrechina*; ‘Forest Opportunists’).

### Statistical Analyses

Our dataset was a site-by-species abundance matrix, with frequency of occurrence in traps (up to 20 per site) used as the measure of abundance. A distribution map of native and exotic ant species at the study scale was produced in ArcMAP 10 [49]. Trapping success (percentage of traps occupied by ants) and native ant richness were compared between rainforest and open habitat sites using non-parametric permutation tests (either exact test, or the asymptotic z-test approximation in case of high permutation number). We investigated how habitat variables were associated with the five most abundant exotic species through Canonical Correspondence Analysis (CCA), with log-transformed and normalised habitat variables [50], using the ‘vegan’ statistical package in R 2.15.0 [51]. Litter depth and litter cover were substantially correlated (Pearson’s r = 0.61; *P*<0.001), so we only kept the former, as recommended by ter Braak [50]. The significance of each canonical axis formed by the linear combination of environmental variables was obtained by permutation (function ‘anova.cca’) [52]. We then investigated patterns of community composition at species, genus and functional group levels in relation to habitat and invasion by ordinating sites using multi-dimensional scaling (MDS), based on Bray-Curtis similarity. One-way analysis of similarity (ANOSIM) was used to investigate pair-wise differences between habitat types (using an *a priori* grouping). We used cluster analysis (CLUSTER procedure with SIMPROF test of significance, 999 permutations) to investigate patterns of native ant assemblages in relation to the abundance of exotic species, independently of vegetation type (without any *a priori* grouping). To assess the extent to which invasive species modify native ant community composition, we ordinated sites with MDS using only native ants, and including rainforest sites only, as other habitats harboured few native species. We also excluded five rainforest sites that were dominated by exotic ants and had no native ants (F17 - F21). We used ANOSIM to assess if native ant composition differed between invaded and non-invaded sites. The extent to which the observed pattern could be explained by habitat variables was assessed with the RELATE procedure. This performs a non-parametric Mantel test between the biotic and abiotic similarity matrices (the latter using Euclidean distances on log-transformed and normalised values), and is based on a Spearman rank correlation coefficient *ρ* (*ρ* = 0 indicates no relation and *ρ* = 1 indicates a perfect match), computed through randomisation tests (999 permutations). To identify native species, genera or functional groups contributing most to the observed dissimilarity between invaded and non-invaded sites, we performed an analysis of similarity percentage (SIMPER). These analyses were conducted using the software PRIMER 6 [53]. Finally, we looked at the relationship between *W. auropunctata* abundance and native ant richness and abundance at rainforest sites, excluding those sites where other Dominant Opportunists occurred.

## Results

**Figure 2:**
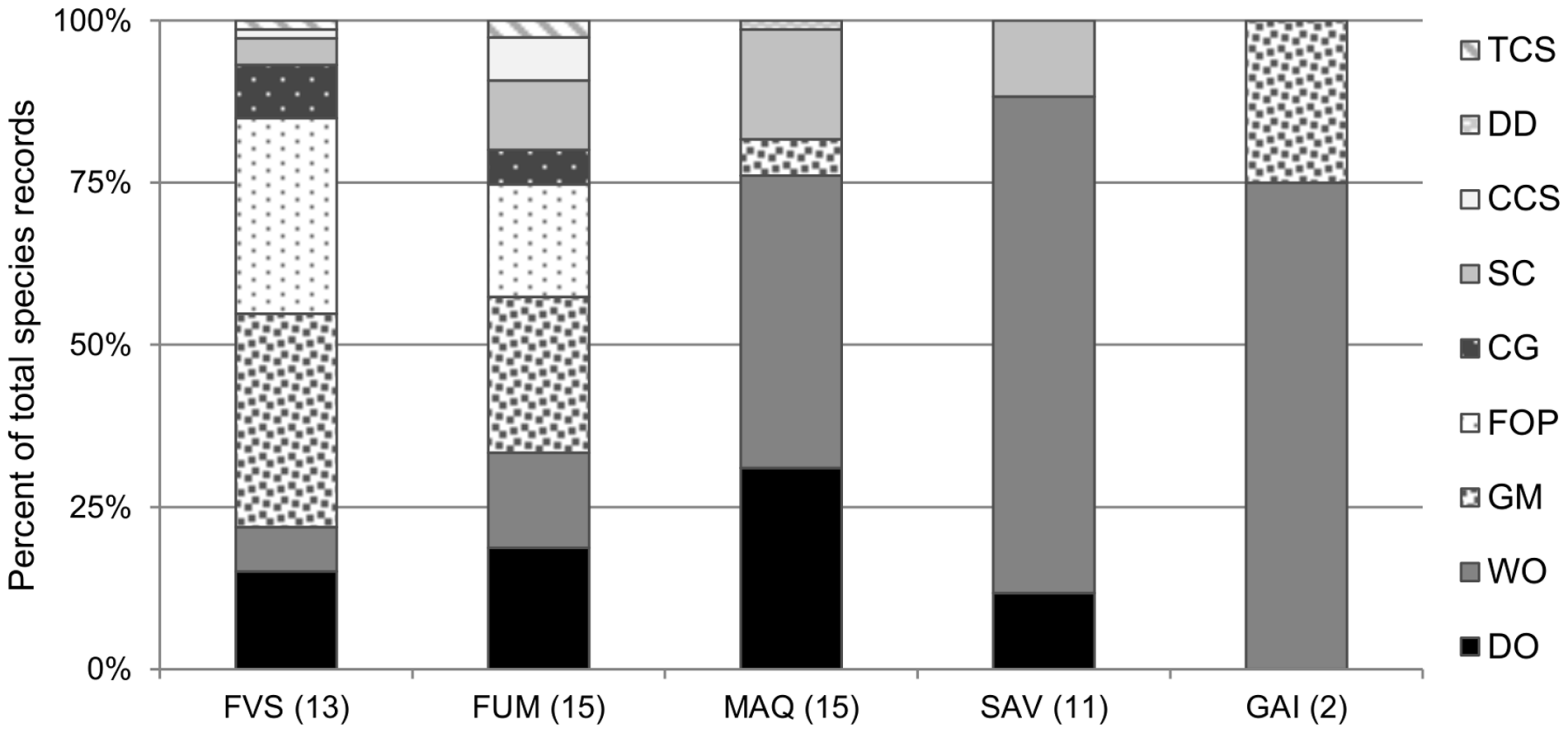
Functional group composition of each habitat type. Values are proportions of total species records. FVS: rainforest on volcano-sedimentary substrate; FUM: rainforest on ultramafic substrate; MAQ: maquis shrubland; SAV: savanna; GAI: gaiac thicket. See Table S2 for the functional group classification.

We recorded 49 species from 20 genera over the 56 sites sampled, with the richest genera being *Pheidole* (8 species), *Rhytidoponera* (6), and *Paraparatrechina* (4) (Table 1). The rainforest sites had lower mean trapping success than open sites (61% ±4 SE *vs* 81% ±4 SE respectively; permutation test using asymptotic approximation Z = −3.41; *P*<0.001), but their mean native species richness was nine times higher (3.6±0.6 SE *vs* 0.4±0.1 SE respectively; exact permutation test *P*<0.001). Functional group composition varied markedly between habitat types (Fig. 2). Dominant Opportunists were present in all habitat types (ranging from 13% of all ants in savanna to 37% in maquis), except in gaiac thicket. The two rainforest types had near identical functional group composition, with Generalized Myrmicinae and Forest Opportunists being the two most abundant groups in both cases. The open habitats had much fewer functional groups, and were dominated numerically by Weedy Opportunists, overwhelmingly so in savanna and gaiac vegetation types.

**Table 1:** Ant species collected and percentage of sites occupied per habitat type.

Species	Functional group	FVS	FUM	MAQ	SAV	GAI
Anoplolepis gracilipes* (Smith)	DO	0	13	80	9	0
*Brachymyrmex* cf. obscurior* (Forel)	WO	0	7	80	100	100
*Brachymyrmex* sp. B*	WO	8	20	13	0	0
*Camponotus* sp. A	SC	15	20	0	0	0
*Camponotus* sp. K	SC	8	0	0	0	0
Cardiocondyla emeryi* (Forel)	WO	8	0	20	18	50
*Dolichoderus* sp. A	CCS	0	13	0	0	0
*Iridomyrmex neocaledonica* (Emery)	DD	0	0	7	0	0
*Leptomyrmex nigriceps* (Emery)	FOP	8	7	0	0	0
*L. pallens* (Emery)	FOP	54	27	0	0	0
*Lordomyrma* sp. Q (*caledonica* gp.)	TCS	8	0	0	0	0
*Lordomyrma* sp. R (*caledonica* gp.)	TCS	0	13	0	0	0
Monomorium floricola* (Jerdon)	WO	0	0	7	0	0
*Monomorium* sp. AA (*pallipes* gp.)	CCS	0	13	0	0	0
*Monomorium* sp. AB (*pallipes* gp.)	CCS	0	7	0	0	0
*Monomorium* sp. X (*antarcticum* gp.)	CCS	8	0	0	0	0
Nylanderia vaga* (Forel)	WO	23	27	80	36	0
*Ochetellus* cf. *glaber* (Mayr)	WO	0	0	7	0	0
*Odontomachus* cf. *simillimus* (Smith)	WO	0	0	0	9	0
*Paraparatrechina* sp. A	FOP	15	0	0	0	0
*Paraparatrechina* sp. C	FOP	8	7	0	0	0
*Paraparatrechina* sp. J	FOP	0	13	0	0	0
*Paraparatrechina* sp. K	FOP	8	0	0	0	0
Paratrechina longicornis* (Latreille)	WO	0	0	0	36	0
Pheidole megacephala* (Fabricius)	DO	46	27	0	0	0
*Pheidole* sp. 1 (*umbonata* gp.)	GM	15	0	0	0	0
*Pheidole* sp. 2 (*umbonata* gp.)	GM	23	47	0	0	0
*Pheidole* sp. 3 (*umbonata* gp.)	GM	0	13	0	0	0
*Pheidole* sp. A	GM	15	20	0	0	0
*Pheidole* sp. B (*umbonata* gp.)	GM	15	0	0	0	0
*Pheidole* sp. C (*umbonata* gp.)	GM	46	20	0	0	50
*Pheidole* sp. D	GM	8	13	7	0	0
*Pheidole* sp. M (*umbonata* gp.)	GM	54	0	7	0	0
*Pheidole* sp. N (*umbonata* gp.)	GM	0	7	0	0	0
*Pheidole* sp. V (*umbonata* gp.)	GM	8	0	0	0	0
*Pheidole* cf. *oceanica* (Mayr)	GM	0	0	13	0	0
*Polyrhachis guerini* (Roger)	SC	0	0	7	9	0
*Rhytidoponera numeensis* (Andre)	FOP	23	0	0	0	0
*R. wilsoni* (Brown)	FOP	0	7	0	0	0
*Rhytidoponera* sp. H (*fulgens* gp.)	FOP	23	0	0	0	0
*Rhytidoponera* sp. I (*fulgens* gp.)	FOP	15	27	0	0	0
*Rhytidoponera* sp. J (*pulchella* gp.)	FOP	8	0	0	0	0
*Rhytidoponera* sp. K (*acanthoponeroides* gp.)	FOP	8	0	0	0	0
Solenopsis geminata* (Fabricius)	DO	0	0	7	9	0
*Solenopsis* sp. B	CG	46	27	0	0	0
Tapinoma melanocephalum* (Fabricius)	WO	0	20	0	9	0
Tetramorium bicarinatum* (Nylander)	WO	0	0	0	9	0
Tetramorium simillimum* (Smith)	WO	0	0	0	9	0
Wasmannia auropunctata* (Roger)	DO	38	53	67	27	0

FVS: rainforest on volcano-sedimentary soils (13 sites); FUM: rainforest on ultramafic soils (15); MAQ: maquis shrubland (15); SAV: savanna (11) and GAI: gaiac thicket (2).

An asterisk indicates an introduced species.

See Table S2 for the functional group classification.

### Exotic Ants’ Habitat

**Figure 3:**
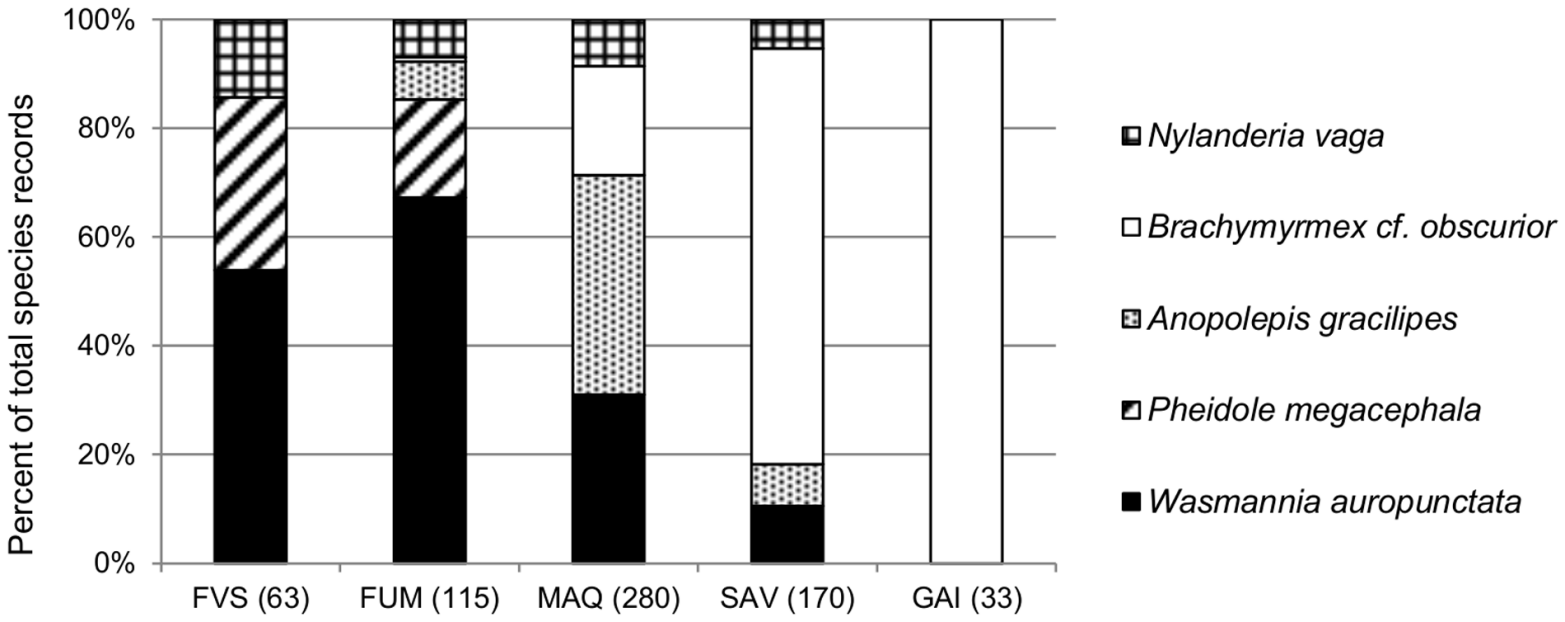
Relative abundance of the five most abundant exotic species according to habitat type. Values are proportions of total records of the five species per habitat type (total records in brackets). FVS: rainforest on volcano-sedimentary substrate; FUM: rainforest on ultramafic substrate; MAQ: maquis shrubland; SAV: savanna; GAI: gaiac thicket.

Thirteen (27%) species were exotic, with the most common being *Brachymyrmex* cf. *obscurior* (220 traps occupied from 26 sites), *W. auropunctata* (217 traps from 26 sites), *A. gracilipes* (134 traps from 15 sites), *Nylanderia vaga* (50 traps from 23 sites), and *P. megacephala* (41 traps from 10 sites). Exotic species classified as Dominant Opportunists occurred at most (71%) sites, and were particularly abundant in the south of the island (Fig. 1b). Only 10% of sites were completely free of exotic species, and these were all rainforests. *Wasmannia auropunctata* and *N. vaga* were both very widespread, occurring in all habitat types except gaiac thicket (Fig. 3). In contrast, *P. megacephala* was restricted to forests, and *B.* cf. *obscurior* was restricted to non-forest habitats, being particularly abundant in gaiac and savanna vegetation types. *Anoplolepis gracilipes* occurred primarily in maquis, but was also found in savannas and rainforests on ultramafic substrate. *Wasmannia auropunctata* and *A. gracilipes* co-occurred at 8 sites (7 in maquis and 1 in rainforest on ultramafic substrate), without a consistent pattern in their relative abundance (paired permutation test T_7_ = 18; *P*>0.05). *Wasmannia auropunctata* and *P. megacephala* co-occurred at two rainforest sites, and *A. gracilipes* and *P. megacephala* co-occurred at one rainforest site.

**Figure 4:**
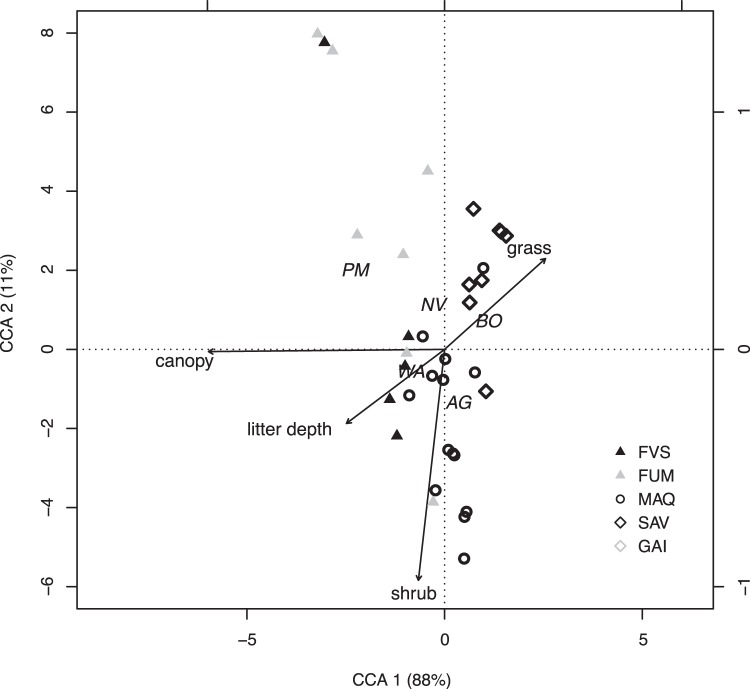
Relationship between habitat variables and the five most abundant exotic species. Canonical Correspondence Analysis triplot of the habitat variables in relation to sites [FVS: rainforests on volcano-sedimentary substrate (10); FUM: rainforests on ultramafic substrate (12); MAQ: maquis-shrubland (14); SAV: savannas (11); GAI: gaiac thickets (2)] and exotic species (AG: *A. gracilipes*; WA: *W. auropunctata*; BO: *B.* cf. *obscurior*; NV: *N. vaga*; PM: *P. megacephala*). Proportion explained by each eigenvalues is reported next to the axis label. The right- and upper axes relate to the environmental variables constraints.

The contrasting habitat preferences of the major exotic species are further illustrated by CCA (Fig. 4; Table 2), with the first two axes explaining 99% of the variance. Only the first axis was found to be significant (Pseudo-F_1,44_ = 13.47; *P*<0.005), and related to habitat openness. *Pheidole megacephala* was strongly associated with a dense canopy (i.e. rainforest habitat), whereas *A. gracilipes* and *B.* cf. *obscurior* showed a contrasting association with open habitats, and, especially in the case of *B.* cf. *obscurior*, grassy habitats. *Wasmannia auropunctata* favoured intermediate levels of canopy cover (as did *N. vaga*) and litter depth. *Anoplolepis gracilipes* showed a preference for shrubby habitat.

**Table 2:** Species scores on the first two CCA components (explaining 88% and 11% of variance, respectively).

	CCA 1	CCA 2
*A. gracilipes*	0.25	−0.34
*B.* cf. *obscurior*	0.78	0.18
*N. vaga*	−0.21	0.29
*W. auropunctata*	−0.60	−0.14
*P. megacephala*	−1.61	0.51

### Native Ant Communities in Relation to Invasion

**Figure 5:**
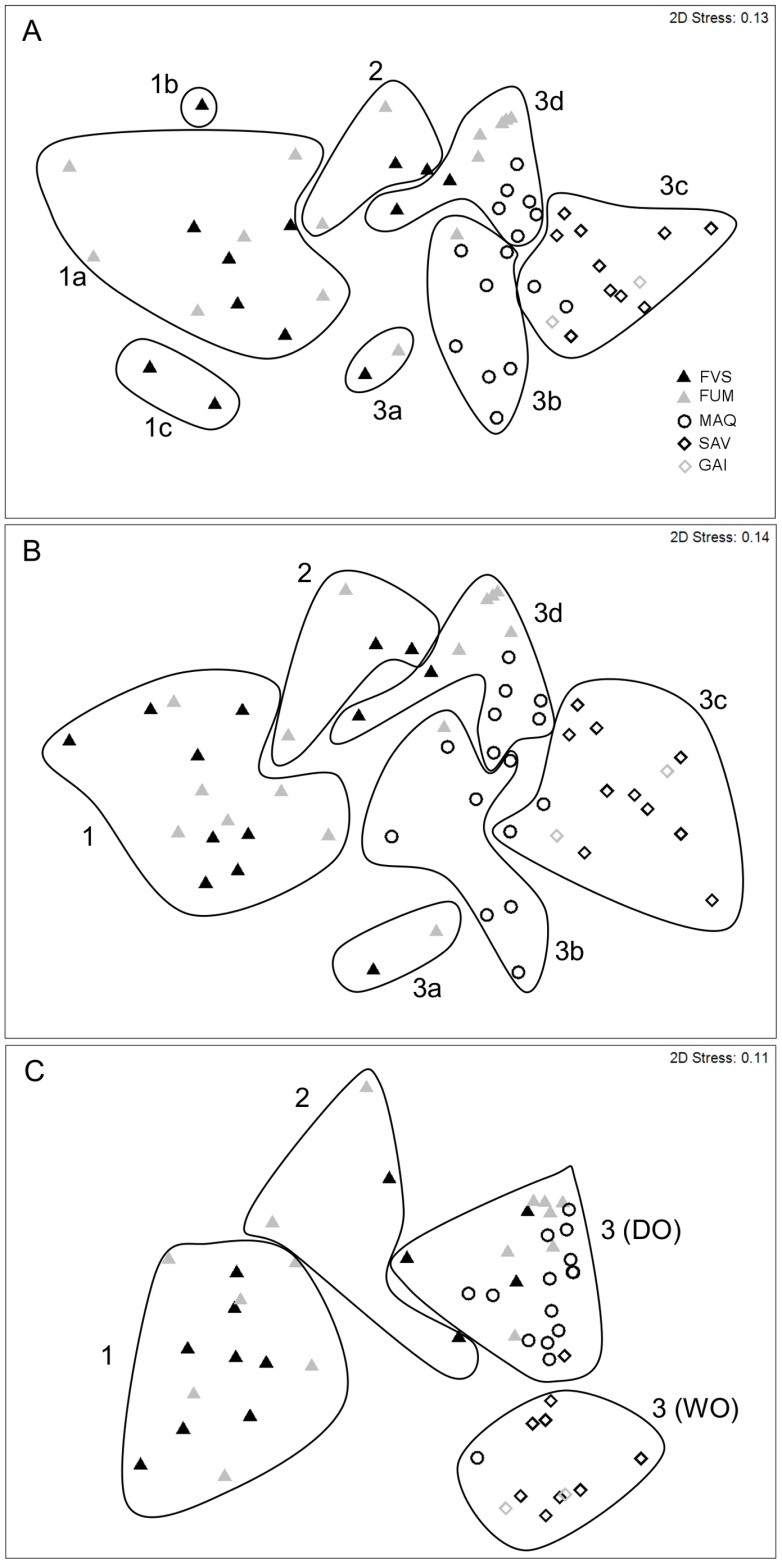
MDS ordinations of sites using different levels of ant community organisation. The significant groups identified by CLUSTER analysis are overlaid: (a) at the species level, (b) at the genus level, (c) at the functional group level. Group 1 (incl. 1a, 1b and 1c): ‘pristine’ forest sites; group 2: forest sites dominated by *Solenopsis* sp. B; group 3: sites dominated by exotic ants (3a: *P. megacephala*; 3b: *A. gracilipes*; 3c: *B.* cf. *obscurior* and 3d: *W. auropunctata*). DO: Dominant Opportunists and WO: Weedy Opportunists. Sites are displayed according to habitat type [FVS: rainforest on volcano-sedimentary substrate (13); FUM: rainforest on ultramafic substrate (15); MAQ: maquis shrubland (15); SAV: savanna (11); GAI: gaiac thicket (2)]. Stress values <0.2 indicate a good 2-D summary of the sample relationships.

MDS based on ant species composition with *a priori* grouping into habitat types showed clear differentiation between forest sites on one hand, and open habitats (maquis, savanna and gaiac sites) on the other (ANOSIM global *R* = 0.417, *P*<0.001; pairwise tests all significant except FVS, FUM *R* = 0.061 *P* = 0.13 and SAV, GAI *R* = −0.32 *P* = 0.90). Cluster analysis without *a priori* grouping revealed that groups identified at the species level were maintained at the genus and functional group levels (Fig. 5a, b and c respectively). Group 1 consisted of the ‘pristine’ forest sites harbouring few or no exotic ants (either Dominant Opportunists or exotic Weedy Opportunists). The species level ordination revealed two small sub-clusters in Group 1: 1b, a single, particularly rich forest site (13 native species, most of which were unique to this site); and 1c, which encompassed the only two forests that recorded *Rhytidoponera* sp. H (Fig. 5a). Group 2 consisted of four forest sites where *Solenopsis* sp. B was particularly abundant and associated with *W. auropunctata* in three of the four sites and with very few other native species. Group 3 contained all the savanna, maquis and gaiac sites, along with the several forest sites that harboured exotic ants in more than one trap. There were several sub-clusters: 3a, containing two forest sites dominated (88% and 95% traps occupied) by *P. megacephala*; 3b, containing seven maquis and one forest site, all dominated (58–100% trap occupancy) by *A. gracilipes*; 3c, containing all savanna and gaiac sites along with two maquis sites, all dominated (50 - 100% trap occupancy) by *B.* cf. *obscurior*; and 3d, containing a range of maquis and forest sites, all dominated (31 - 100% trap occupancy) by *W. auropunctata*. The sub-clusters of group 3 were similar in the functional group ordination (Fig. 5c), with one cluster characterized by Dominant Opportunists (previously Group 3a, 3b, and 3d, plus one maquis site and one savanna site previously in Group 3c) and another by Weedy Opportunists (Group 3c).

**Figure 6:**
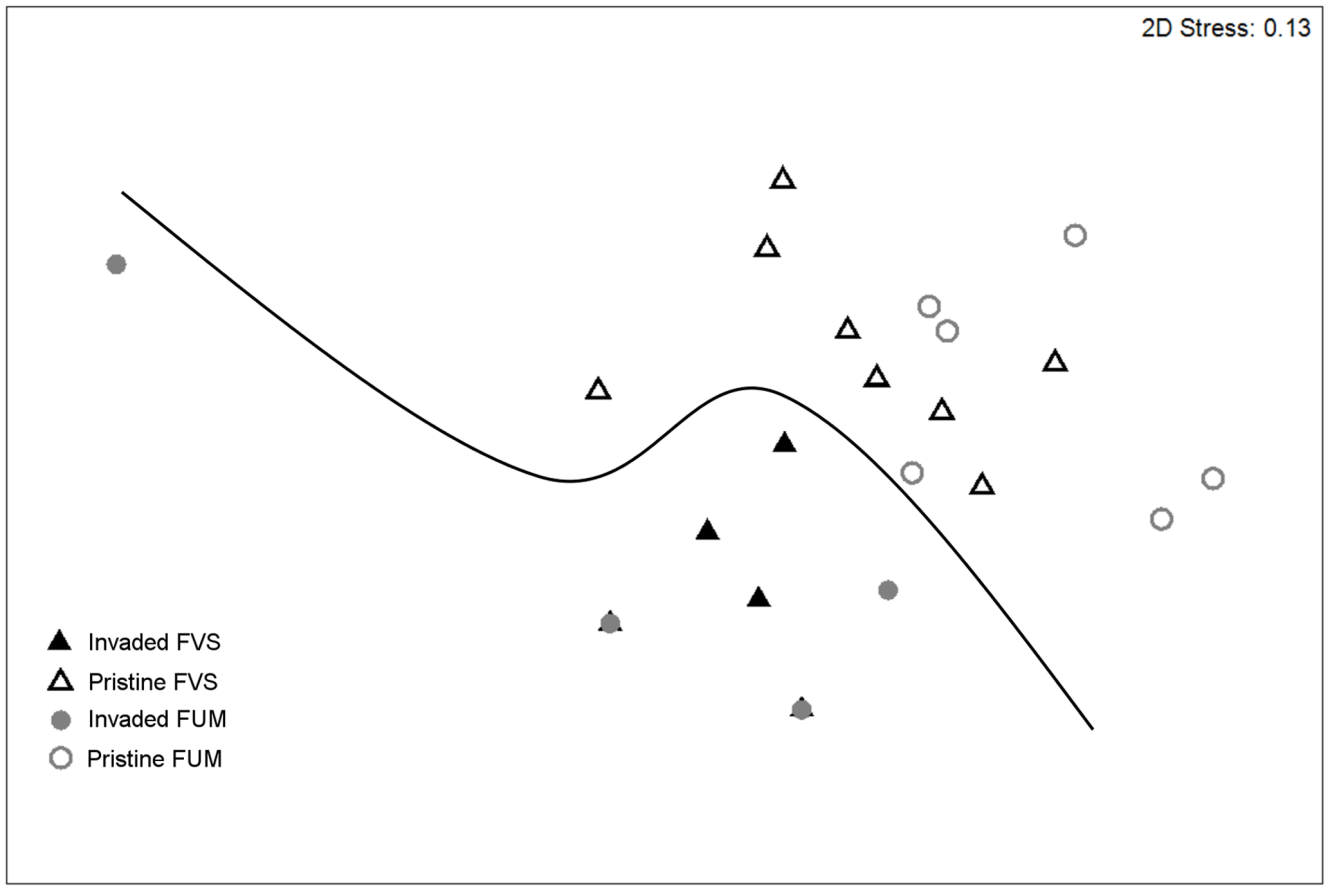
MDS ordination of rainforest sites based on native ants only, at the species level. The significant difference identified by ANOSIM between invaded and non-invaded sites is indicated by a line. FVS: rainforest on volcano sedimentary substrate; FUM: rainforest on ultramafic substrate. Stress values <0.2 indicate a good 2-D summary of the sample relationships.

When excluding exotic species and considering rainforest sites only (n  = 23), we found a significant difference in community composition between sites that belonged to the ‘pristine’ group (Group 1) and sites that were dominated by exotic species in the previous analysis (Groups 2 and 3). This was true at the species (ANOSIM global *R* = 0.53, *P*<0.01; Fig. 6), genus (ANOSIM global *R* = 0.70, *P*<0.001) and functional group (ANOSIM global *R* = 0.71, *P*<0.001) levels, and was independent of the substrate type (ANOSIM *P*>0.05 in all cases). The RELATE procedure revealed no significant relationship between the similarity matrices of ant community composition and of habitat variables (*ρ* = 0.07; *P* = 0.26). SIMPER revealed that Generalized Myrmicinae and Forest Opportunists were the functional groups contributing most (34 and 30% respectively) to the dissimilarity (Table 3). *Leptomyrmex pallens* was the species that contributed most to the dissimilarity between invaded and pristine sites, being highly associated with the latter (11.2% contribution; Table 3), whereas *Solenopsis* sp. B was positively associated with invasion (22.6% contribution; Table 3). Finally, we found a negative relationship between the abundance of *W. auropunctata* and native ant species richness (*R^2^* = 0.50; *P*<0.001) and abundance (*R^2^* = 0.50; *P*<0.001) at rainforest sites. *Pheidole megacephala* occurred at ten rainforest sites, and was the only Dominant Opportunist in seven of these. It was extremely abundant in two of these sites (75 and 90% respectively) and the only native ant species that was present at these sites was *Solenopsis* sp. B.

**Table 3:** Contribution of native ants to the dissimilarity between ‘pristine’ rainforest sites and rainforest sites dominated by exotic species, with exotic species excluded from the dataset.

Organisational level	‘Pristine’	Dominated by exotic species
**Species**	*L. pallens* (11.2%)	*Solenopsis* sp. B (23.9%)
	*Pheidole* sp. 2 (9.8%)	
	*Pheidole* sp. M (6.4%)	
	*Pheidole* sp. C (5.9%)	
	*Rhytidoponera* sp. H (5.1%)	
	*Rhytidoponera* sp. I (4.9%)	
	*Pheidole* sp. A (4.8%)	
**Genus**	*Pheidole* (33%)	*Solenopsis* (25%)
	*Rhytidoponera* (15%)	
**Functional group**	GM (34%)	CG (26%)
	FOP (30%)	

Contributions up to a 70% cumulative cut-off value are indicated, using the SIMPER procedure.

GM: generalised myrmicinae, FOP: forest opportunists, CG: cryptic generalists.

## Discussion

Our broad-scale survey shows that none of New Caledonia’s main habitat types are free of exotic species, with only a small number of our rainforest sites being uninvaded. We recorded five particularly abundant species, three of which are listed among the world’s worst invasive species (Lowe et al. 2000): *W. auropunctata*, *A. gracilipes* and *P. megacephala.* The other two were *B.* cf. *obscurior* and *N. vaga*, both widely spread in the Pacific region [54] but not considered to be a major ecological threat. Unsurprisingly, we found exotic ants to be particularly abundant in disturbed, open habitats (savanna, maquis and gaiac), and here native ant species were virtually absent. However, exotic ants also occurred in undisturbed rainforest.

The most widespread exotic species was *W. auropunctata*, which was most abundant in rainforests, and to a lesser extent in maquis and savannas. Its preference for moderate levels of canopy cover and litter development is consistent with results from previous studies in New Caledonia and elsewhere [17], [55]–[57]. *Nylanderia vaga* showed a similar habitat association, but limited literature can be found on its habitat preference elsewhere [9], [42]. *Anoplolepis gracilipes* was strongly associated with maquis shrubland, with high abundance at 12 of the 15 maquis sites. Apart from one site where it was abundant but co-occurring with *W. auropunctata*, it was virtually absent from rainforests, and so our findings suggest that it has little direct impact on the New Caledonian native ant fauna (i.e. it is a ‘passenger’ of disturbance, see MacDougall and Turkington [58]). This is in stark contrast to its distribution and impacts elsewhere, where it can be highly abundant in rainforest [2], [59], and indicates that it is possibly still in a latency phase of invasion in New Caledonia [60]. *Pheidole megacephala* was restricted to urban areas in New Caledonia in the early stages of its invasion [28], when it might also have been in a latency, but was only found in rainforest in our study, and was associated with very low native ant richness at sites where it was abundant. The capacity of the species to settle in and dominate degraded habitat, as well as pristine rainforests throughout its invaded range is well documented [7], [61]–[64]. The weedy species *B.* cf. *obscurior* was by far the most frequently recorded species (contributing to 23% of all species records), but was restricted to open habitats and gaiac (*A. spirorbi*s) thickets. This is consistent with its presence in disturbed, open habitats throughout its introduced range [65], [66]. The extremely high abundance of *Brachymyrmex* cf. *obscurior*, in particular in gaiac thickets from which *W. auropunctata* was absent, could be explained by its capacity to compete successfully with the latter in one-on-one interactions [28], and its high ability to exploit honeydew resources [28], [67].

Variation in overall ant species composition was strongly associated with the distribution and abundance of exotic species, with most sites falling into one of two groups: either ‘pristine’ forest sites with no exotic species (Group 1), or sites dominated by exotic species and representing all habitat types (Group 3). A small number of sites comprised a third group (Group 2) characterised by a high abundance of *Solenopsis* sp. B, mostly co-occurring with *W. auropunctata*. *Solenopsis* sp. B was particularly abundant at rainforest sites where native ant diversity was low; this could be an indication that *Solenopsis* sp. B is in fact an exotic species, possibly *S. papuana*, reported by Jourdan (1999) as occurring in New Caledonia, and predominantly occurring in rainforest habitat (http://www.antweb.org/).

The above patterns were maintained when analyses were repeated at the genus and functional group levels. The congruence between species and genus compositional patterns can be explained by the strong correlation between species and genus richness at our study sites (Fig. S1), as suggested in Andersen [68]. The maintenance of these patterns with analysis at the functional group level demonstrates strong congruence between taxonomic and functional composition. However, our methodology sampled only a limited range of functional groups, largely overlooking specialised taxa such as cryptic species and specialist predators, and more comprehensive sampling may lead to a more composite outcome.

The composition of native ants at species, genus and functional group levels was different at invaded compared with uninvaded rainforest sites, with no indication of habitat (abiotic variables nor substrate) playing a significant role. We did not experimentally test the respective roles of habitat disturbance and invasion in shaping native ant communities [58], [69], but the absence of systematic habitat difference between invaded and non-invaded rainforests underlines the importance of invasion *per se*. This is consistent with a recent study showing that both *W. auropunctata* and *A. gracilipes* have direct impacts on native ants communities [70]. The negative relationship between the abundance of *W. auropunctata* and native ant abundance and richness further illustrates that, in addition to dominating many ant communities numerically, exotic species, and in particular *W. auropunctata*, alter native ant communities. A loss of Forest Opportunists and Generalized Myrmicinae was characteristic of invaded sites, making these groups suitable indicators of invasion. In contrast to Cryptic Generalists, which forage within the litter and are often reported to persist in invaded habitats [22], [56], [71], these groups overlap with *W. auropunctata*’s and *P. megacephala*’s epigaeic foraging strata.

Our results corroborate the finding that, while considered as a ‘disturbance specialist’ in its natural range [72], [73], *W. auropunctata* has the ability to dominate and negatively impact the native ant fauna in undisturbed rainforest in its introduced range [22].

## Supporting Information

Figure S1 **Relationship between species and log-transformed genus richness of native ants.** Only the 30 sites where native ants were recorded are included (Y = −3.32+6.1 X; adjusted *R^2^* = 0.81; *P*<0.001). Residuals conformed to linear model assumptions.
(TIF)

Table S1 **List of sampled sites. F: rainforest; MAQ: maquis-shrubland; SAV: savanna; GAI: gaiac (** ***Acacia spirorbis*** **) thicket. VS: volcano-sedimentary substrate; U: ultramafic substrate.** The legal sampling permits including ant samples were provided by the both Environmental Departments of the Northern and Southern Provinces (EDNP and EDSP, respectively), and where appropriate, oral authorization was also obtained from tribes and private owners when sampling areas were also located on tribal lands.
(DOCX)

Table S2 **Functional group classification of New Caledonian ants, modified from the more general scheme described by Andersen (1995a).**
(DOCX)
