# Supplementary material for: Overview of the Distribution, Habitat Association and Impact of Exotic Ants on Native Ant Communities in New Caledonia
Source: PLoS One. 2013 Jun 26;8(6):e67245. doi: 10.1371/journal.pone.0067245 (PMC3693956; doi:10.1371/journal.pone.0067245)
Supplement: Table S1 — List of sampled sites. F: rainforest; MAQ: maquis-shrubland; SAV: savanna; GAI: gaiac ( Acacia spirorbis ) thicket. VS: volcano-sedimentary substrate; U: ultramafic substrate. The legal sampling permits including ant samples were provided by the both Environmental Departments of the Northern and Southern Provinces (EDNP and EDSP, respectively), and where appropriate, oral authorization was also obtained from tribes and private owners when sampling areas were also located on tribal lands. (DOCX) [file pone.0067245.s002.docx]

Table S1

| Plot # | Latitude (dec) | Longitude (dec) | Altitude (m) | Vegetation type | Substrate | Authorization source / tribe or sector |
| --- | --- | --- | --- | --- | --- | --- |
| F01 | 164.56833 | -20.40861 | 83 | F | VS | EDNP/ Pouébo tribe |
| F02 | 164.73935 | -20.68576 | 519 | F | VS | EDNP / Bas-Coulna tribe |
| F03 | 164.73407 | -20.6807 | 523 | F | VS | EDNP / Bas-Coulna tribe |
| F04 | 164.71864 | -20.66719 | 634 | F | VS | EDNP / Bas-Coulna tribe |
| F05 | 164.7192 | -20.66701 | 640 | F | VS | EDNP / Bas-Coulna tribe |
| F06 | 164.88853 | -20.99517 | 380 | F | VS | EDNP / Ateu tribe |
| F07 | 164.8955 | -20.98011 | 476 | F | VS | EDNP / Ateu tribe |
| F08 | 164.91034 | -20.94028 | 583 | F | VS | EDNP / Atéou tribe |
| F09 | 165.91076 | -20.94136 | 591 | F | VS | EDNP / Atéou tribe |
| F11 | 165.81467 | -21.61498 | 538 | F | VS | EDSP / RP5 road sector Grand Couli |
| F12 | 165.82876 | -21.57179 | 368 | F | VS | EDSP / RP5 road sector Grand Couli |
| F13 | 166.66537 | -22.21362 | 262 | F | UM | EDSP / RP3 road sector Col de Mouirange – Col de deux tétons |
| F14 | 166.8931 | -22.16918 | 312 | F | UM | EDSP / RP3 road near Yaté village |
| F15 | 166.89612 | -22.1688 | 368 | F | UM | EDSP / RP3 road near Yaté village |
| F16 | 166.93633 | -22.15968 | 30 | F | UM | EDSP / RP3 road near Yaté village |
| F17 | 166.93012 | -22.15892 | 23 | F | UM | EDSP / RP3 road near Yaté village |
| F18 | 166.91271 | -22.16457 | 253 | F | UM | EDSP / RP3 road near Yaté village |
| F19 | 166.91172 | -22.16272 | 283 | F | UM | EDSP / RP3 road near Yaté village |
| F20 | 166.71556 | -22.3015 | 39 | F | UM | EDSP / Prony’s trail sector Mont-Dore |
| F21 | 166.71627 | -22.30147 | 55 | F | UM | EDSP / Prony’s trail sector Mont-Dore |
| F22 | 165.81405 | -21.58705 | 357 | F | VS | EDSP / RP5 road |
| F23 | 165.81979 | -21.53191 | 204 | F | VS | EDNP / RPN3 road sector Koh-Kouaoua |
| F25 | 166.3317 | -22.06925 | 470 | F | UM | EDSP / “col de la pirogue” private wellness center |
| F26 | 166.33085 | -22.07065 | 433 | F | UM | EDSP / “col de la pirogue” private wellness center |
| F27 | 166.33003 | -22.07175 | 404 | F | UM | EDSP / “col de la pirogue” private wellness center |
| F28 | 166.33257 | -22.0755 | 401 | F | UM | EDSP / sector “col de la pirogue” |
| F29 | 166.33413 | -22.07569 | 441 | F | UM | EDSP / sector “col de la pirogue” |
| F30 | 166.50973 | -22.1756 | 518 | F | UM | EDSP / sector Mt Koghi Fern Forest |
| M01 | 166.66622 | -22.21467 | 218 | MAQ | UM | EDSP /RP3 road sector “Col de Mouirange – Col de deux tétons |
| M02 | 166.87231 | -22.15929 | 185 | MAQ | UM | EDSP /RP3 road  near Yaté lake |
| M03 | 166.8618 | -22.16698 | 186 | MAQ | UM | EDSP /RP3 road  near Yaté lake |
| M04 | 166.64994 | -22.22473 | 177 | MAQ | UM | EDSP /RP3 road, sector “la Coulée – col de Mouirange” |
| M05 | 166.64901 | -22.22459 | 167 | MAQ | UM | EDSP /RP3 road, sector “la Coulée – col de Mouirange” |
| M06 | 166.75792 | -22.14557 | 162 | MAQ | UM | EDSP / RP3 road,  near Yaté lake |
| M07 | 166.7589 | -22.14637 | 163 | MAQ | UM | EDSP / RP3 road,  near Yaté lake |
| M08 | 166.83644 | -22.17442 | 192 | MAQ | UM | EDSP / Madeleine Waterfall road  near Yaté lake |
| M09 | 166.83759 | -22.17317 | 193 | MAQ | UM | EDSP / /Madeleine Waterfall road  near Yaté lake |
| M10 | 166.69692 | -22.28313 | 40 | MAQ | UM | EDSP / Southern road  sector Pirogues’ river |
| M11 | 166.69876 | -22.28139 | 39 | MAQ | UM | EDSP / Southern road  sector Pirogues’ river |
| M12 | 166.7854 | -22.30746 | 170 | MAQ | UM | EDSP /Prony trail road |
| M13 | 166.78355 | -22.30852 | 175 | MAQ | UM | EDSP /Prony trail road |
| M14 | 166.81106 | -22.31265 | 167 | MAQ | UM | EDSP /sector Prony village |
| M15 | 166.81148 | -22.31395 | 163 | MAQ | UM | EDSP /sector Prony village sector |
| S01 | 164.18665 | -20.29298 | 58 | SAV | VS | EDNP / RPN9 road near RPN1 |
| S02 | 164.18416 | -20.29572 | 78 | SAV | VS | EDNP / RPN9 road near RPN1 |
| S03 | 164.19462 | -20.36775 | 24 | SAV | VS | EDNP / RPN1 road near Nehoue Bay |
| S04 | 164.19456 | -20.36819 | 24 | SAV | VS | EDNP / RPN1 road near Nehoue Bay |
| S05 | 164.2109 | -20.39468 | 20 | SAV | VS | EDNP / RPN1 road near Nehoue Bay |
| S06 | 164.57271 | -20.40233 | 152 | SAV | VS | EDNP / Pouébo tribe |
| S07 | 164.57211 | -20.40161 | 121 | SAV | VS | EDNP / Pouébo tribe |
| S08 | 166.10961 | -21.72519 | 164 | SAV | VS | EDNP / RP4 road |
| S10 | 165.81343 | -21.59636 | 328 | SAV | VS | EDSP / RP5 road sector Col d’Amieu |
| S11 | 165.8366 | -21.56091 | 303 | SAV | VS | EDNP / RP5 road sector Col d’Amieu |
| S12 | 166.37981 | -22.15875 | 50 | SAV | VS | EDSP / near Savannah district |
| G01 | 164.8826 | -21.01589 | 73 | GAI | VS | EDNP / Tiakana tribe |
| G02 | 164.8669 | -21.99705 | 139 | GAI | VS | EDNP / Tiakana tribe |
