# Supplementary material for: Overview of the Distribution, Habitat Association and Impact of Exotic Ants on Native Ant Communities in New Caledonia
Source: PLoS One. 2013 Jun 26;8(6):e67245. doi: 10.1371/journal.pone.0067245 (PMC3693956; doi:10.1371/journal.pone.0067245)
Supplement: Table S2 — Functional group classification of New Caledonian ants, modified from the more general scheme described by Andersen (1995a). (DOCX) [file pone.0067245.s003.docx]

Table S2

| Functional group | Code | Description | Major taxa |
| --- | --- | --- | --- |
| Cold Climate Specialists | CCS | as in Andersen (1995) | *Monomorium* |
| Cryptic Generalists | CG | Small to minute generalists living in soil and litter | *Solenopsis,*  *Carebara*,  *Vollenhovia* |
| Dominant Dolichoderinae | DD | as in Andersen (1995) | *Iridomyrmex* |
| Dominant Opportunists | DO | Invasive species with capacity for dominating resources | *Anoplolepis gracilipes, Wasmannia auropunctata*  *Pheidole megacephala* |
| Forest Opportunists | FOP | Native opportunists strongly associated with rainforest habitat | *Leptomyrmex,*  *Rhytidoponera,*  *Paraparatrechina* |
| Generalised Myrmicinae | GM | as in Andersen (1995) | *Pheidole* |
| Subordinate Camponotini | SC | as in Andersen (1995) | *Camponotus* |
| Tropical Climate Specialists | TCS | as in Andersen (1995) | *Lordomyrma* |
| Weedy Opportunists | WO | Ruderal, often exotic species strongly associated with highly disturbed habitats | *Brachymyrmex,*  *Ochetellus* |
